# Ten simple rules to power drug discovery with data science

**DOI:** 10.1371/journal.pcbi.1008126

**Published:** 2020-08-27

**Authors:** Enrico Ferrero, Sophie Brachat, Jeremy L. Jenkins, Philippe Marc, Peter Skewes-Cox, Robert C. Altshuler, Caroline Gubser Keller, Audrey Kauffmann, Erik K. Sassaman, Jason M. Laramie, Birgit Schoeberl, Mark L. Borowsky, Nikolaus Stiefl

**Affiliations:** 1 Computational Sciences Council, Novartis Institutes for BioMedical Research, Basel, Switzerland; 2 Computational Sciences Council, Novartis Institutes for BioMedical Research, Cambridge, Massachusetts, United States of America; 3 Computational Sciences Council, Novartis Institutes for BioMedical Research, Emeryville, California, United States of America; Dassault Systemes BIOVIA, UNITED STATES

## Introduction

Biomedical research is increasingly a high-dimensional science. In the pharmaceutical industry, data supporting the drug discovery and development process have become cheaper to generate and are increasing in complexity, diversity, and volume at a fast pace. This is the result of the introduction and development of novel technologies that enable molecular profiling, imaging, and other types of high-throughput readouts at an unprecedented scale. Similarly, clinical data created by companies or compiled by biobanks are growing at an exponential pace, not only in terms of sample size but also in the breadth of (increasingly digital) endpoints being measured and captured.

In parallel to the increase in data, methodological advances are driving renewed development in statistical modeling, machine learning, and artificial intelligence (AI). The combination of data, computing power, and advanced analytics is positioning data science as a critical core discipline in pharmaceutical research, alongside the more traditional disciplines of biology, chemistry, and medicine.

**Fig 1:**
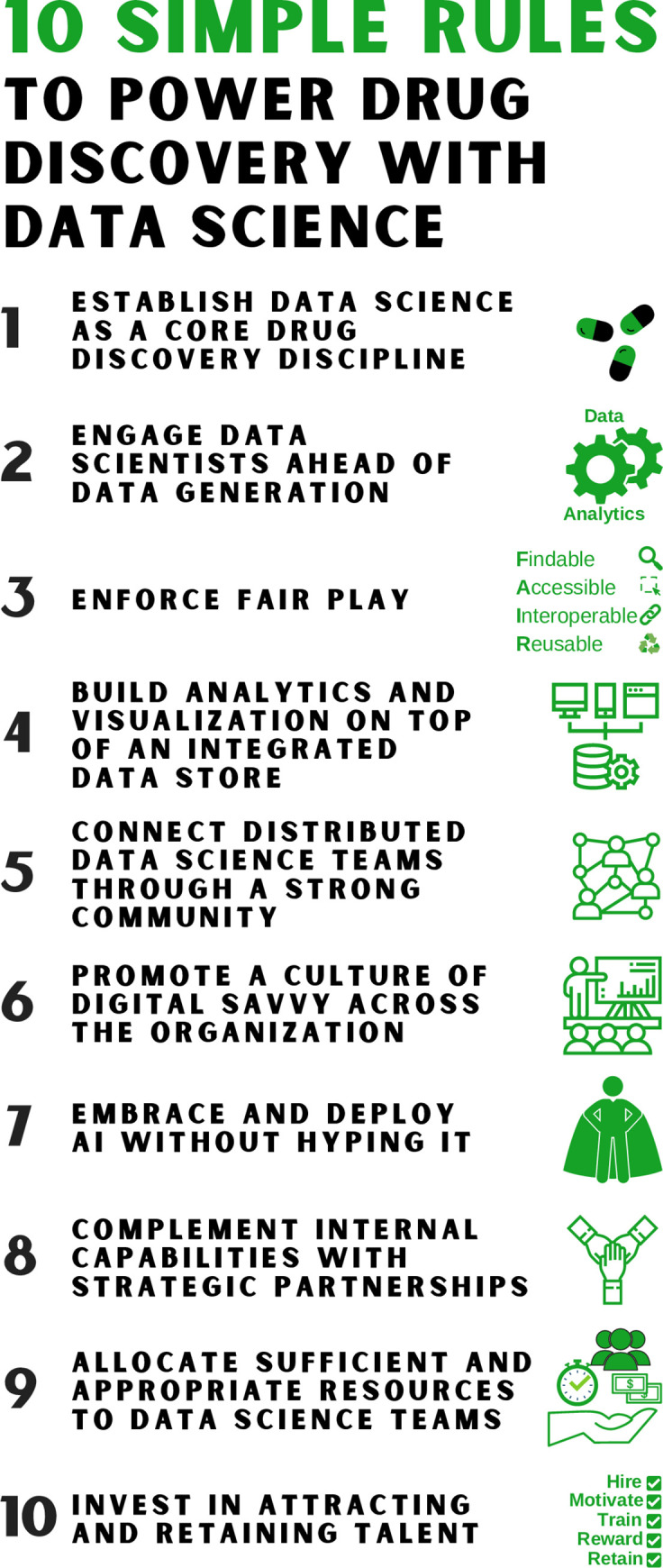
A graphical representation of the ten simple rules to power dug discovery with data science.

Realizing the full potential of data science requires adapting both the structure and culture of an organization. Signs of this transformation can already be seen across the pharmaceutical industry, with the creation of large data science teams and executive roles responsible for the implementation of a company-wide data science strategy. At a time when discovering transformative medicines is more challenging and requires more scientific creativity than ever before, we offer strategic recommendations to those who aspire to propel a digital culture shift and data science transformation in their organizations (Fig 1).

## Rule 1: Establish data science as a core drug discovery discipline

The disciplines of biology, chemistry, and medicine have anchored drug discovery research since its inception; data science is a recent development in comparison. Yet, it is widely recognized that public and proprietary data, together with the ability to extract knowledge from them, are key assets that can drive competitive advantage. In the context of the pharmaceutical industry, data science can be defined as the discipline at the interface of statistics, computer science, and drug discovery. As such, it includes, but it is not limited to, roles such as clinical statisticians, computational chemists, biostatisticians, and computational biologists who have been contributing to drug discovery and development through in silico analyses of large datasets long before the term data science was popularized. More recently, machine learning engineers and specialized data scientists with specific skillsets (e.g., deep learning, image processing, or body sensors analysis) have joined the ranks of growing data science teams in pharmaceutical companies.

While the impact these scientists are having in both early and late drug discovery projects is recognized and often highly visible within an organization, the constituency of senior leadership teams do not necessarily mirror these recent developments in technology and science. In order to establish data science as a core drug discovery discipline, team composition needs to evolve at all levels: from leadership to project teams. Developing a greater understanding within leadership teams of the potential, applications, limitations, and pitfalls of data science in the pharmaceutical industry is now critical. Inclusion of data science leaders in decision-making bodies connects data scientists to critical business questions, raises organizational awareness of computational approaches and data management, and further connects disease-focused departments with discovery and clinical platforms. While the relatively recent emergence of data science means its practitioners may have less extensive career experience in pharmaceutical research than their peers in other functions, they are likely to provide novel perspectives and take orthogonal approaches to the difficult task of discovering and developing new drugs.

Traditional drug discovery project teams are composed of key scientific experts: biologists, pharmacologists, chemists (or antibody engineers), and clinicians, who collaborate to move the programs from target discovery to clinical trials. For projects to be fueled by computational insights and predictions, data scientists need to be integral members of the project teams and engage as collaborators (as opposed to being perceived as just a support function). This enables the development of a project-specific data strategy, deployment of resources required for the more data intensive phases of the program, and application of the most effective computational methods to address the key project questions.

## Rule 2: Engage data scientists ahead of data generation

The generation of high-quality data to support drug discovery activities is of critical importance. No data analysis, regardless of its level of sophistication, can extract valuable insights from low quality data or inadequately designed experiments. As famously pointed out by Ronald Fisher: “To consult the statistician after an experiment is finished is often merely to ask him to conduct a post mortem examination. He can perhaps say what the experiment died of*”* [1]. Yet, too often data analysis is an afterthought, assumed to be easily and quickly plugged in at the end of the experiment without much consideration. Poorly designed experiments not only lead to limited biomedical insights, they also take longer to analyze as data scientists attempt to correct or find workarounds for confounders or other avoidable issues. Experimental designs that take into account best practices and data analysis needs result in more accurate, interpretable, and, therefore, actionable results. They also mean faster analysis times, thus freeing up data science resources for other projects.

In a forward-looking organization that wants to fully realize the potential of data science, data generation and analysis need to be given equal importance. Experimental and computational scientists should work together from the start of projects to design experiments in ways that address the biological questions at hand. Data scientists and experimental scientists should achieve a mutual understanding of one another’s requirements and desired outcomes, something that can be accomplished through regular dialogue and information exchange.

Data are an asset: Novel technologies, lab equipment, academic collaborations, and business partnerships should all be approached with this mindset and quickly complemented with a data management and analysis strategy. Tighter integration of data generation, engineering, and analysis functions also creates shared accountability and improves the impact of combined experimental and computational work supporting drug discovery programs.

Data scientists in the pharmaceutical industry are increasingly tasked with the integration of internal data with the wealth of data available in the public domain. Organizations must consider current external data standards, modeling internally-generated data in compatible formats to enable seamless integration and augmentation of internal knowledge with public data assets. Importantly, projects often do not start with data generation but, rather, with data reuse and integration, so it is essential that new data are natively and fully compatible with existing data.

## Rule 3: Enforce FAIR play

Generating data is resource intensive, and increasing efforts are made in industry and academia to support analysis of data compendia as well as reuse of existing datasets for purposes different from those for which they were originally generated. These efforts are exemplified by the FAIR principles [2,3], which provide guidelines for making data “FAIR”: Findable, Accessible, Interoperable, and Reusable. Retrospective data stewardship to FAIRify existing data is extremely time- and cost-consuming, especially for large, long-established pharmaceutical companies that have accumulated considerable amounts of legacy data, often fragmented across the organization. Over time, important information is lost due to organizational changes and employee turnover, either because the data are not well documented or because they are stored in nonstandard or nonmachine-readable formats. Thus, it is crucial to have in place FAIR play processes from the point of data generation, including clear data and metadata management strategies.

Organizations must set expectations and provide incentives for scientists generating data to include rich and harmonized metadata, and the process to do so should be simple and straightforward. Tools for metadata capture need to be intuitive, flexible, and configurable enough to handle new data types in real time, while adhering to established controlled vocabularies and ontologies. Metadata registration and curation tools should capture identifying, descriptive data and relationships in a single source of truth system and seamlessly propagate information to downstream applications and services. User-friendly study design tools should be integrated with both data production and analysis, providing a platform for experimental scientists and data scientists to collaboratively specify, iterate, and agree upon experimental parameters, analysis methods, and statistical power before running studies.

Importantly, clear data access rules need to be established as part of this FAIRification process. The goal of these rules should be to democratize the data, in which they are no longer accessible by a select few but by the whole organization. This requires a cultural shift away from a “my data” and toward an “our data” mindset. These access policies should clearly establish which data can be accessed by whom and when, with a drive towards reducing bureaucracy and speeding up scientific insights. This is especially valuable in drug discovery and development, during which early and broad access to both preclinical and clinical data may enable the generation of new hypotheses or steer existing programs in new directions. Overall, a FAIR data ecosystem is the foundation from which data scientists select and integrate subsets of data necessary to perform meta-analyses across different datasets.

## Rule 4: Build analytics and visualization on top of an integrated data store

The ability to extract knowledge from data is a primary goal and a key competitive advantage. The challenge is how to find the data of interest, connect them with the linked metadata, and access and analyze the data in order to gain new insights. Novartis started the Nerve Live [4] and data42 [5] projects with these objectives in mind, and many other pharmaceutical companies are actively investing in and working on similar efforts.

Following the FAIR principles is a cornerstone of this challenge, but it is not enough by itself. Fully realizing the value of the data requires developing resources and tools to enable data scientists, experimental scientists, and clinicians to explore, visualize, and analyze data. The ideal toolbox contains a central search engine that indexes all data and lists existing knowledge of key entities and the relationships among them (e.g., targets, compounds, indications, biological pathways, experiments, studies, and portfolio projects), application programming interface APIs to access the data programmatically, and interactive graphical user interfaces (GUIs) to visualize datasets and results. The key is the ability to merge data and query across datasets, conditioned on the use of rich structured metadata and creation of systems that overcome historical data silos. Strategic investments need to be made on data management, data repositories, and FAIR play processes. De novo generation of data to fill crucial data gaps should also be considered.

Another key challenge for large organizations is to maintain nimble systems for exposing the data, while enabling a variety of needs and users, through multiple graphical and programming interfaces. While experimental scientists and clinicians may be more comfortable with dedicated dashboards and reports implemented using platforms such as Shiny [6], Spotfire [7], or Tableau [8], data scientists will expect to interact programmatically with the data through scripting languages such as R [9], Python [10] or SAS [11]. While SAS may be preferred by clinical statisticians and Python has been gaining popularity in recent years, R might still be predominant in the pharmaceutical industry thanks to its strong ties with the bioinformatics community, as exemplified by the popularity of the Bioconductor project [12,13]. Both R and Python offer efficient ecosystems for data wrangling, modeling, and visualization: R as part of the tidyverse [14] and through independent packages like data.table [15], mlr [16,17], and lattice [18] and Python with packages such as pandas [19,20], scikit-learn [21], and matplotlib [22]. Integrating these software solutions with other domain-specific tools is a common challenge for specialists dealing with “-omics” readouts, chemical structures, images, sensor data, clinical, and real-world data. Regardless of the different toolsets in use, one guiding principle for such a system should be to pursue and enforce best practices for reproducible research [23]. Fully recognizing the variety of needs and requirements of data scientists across the organization is critical to ensuring successful design and implementation of a centralized analytics ecosystem.

## Rule 5: Connect distributed data science teams through a strong community

Over the last couple of decades, pharmaceutical companies have experimented with different models to enable effective synergy and integration between data scientists and their collaborators. Adopting the right model is crucial to maximize the impact of data science groups within an organization and is of particular relevance in large pharmaceutical companies, in which the size of the business can become a hindrance to effective collaboration.

In the classical model, a centralized data science group provides support to technology and platform groups as well as to the disease- and program-centric departments. Such a model allows for a great degree of flexibility and efficient resource allocation as data science capabilities can be deployed effectively to different projects and adapted quickly to the fast-evolving needs and priorities of the organization. In such a model, however, complete and successful engagement becomes more difficult as data scientists have limited exposure to the area of research and project specifics. In contrast, a model where distributed computational teams are embedded in each department ensures continued exposure of biomedical data scientists to projects in a well-defined area of research (e.g., oncology, autoimmunity, target discovery, chemistry, or drug safety) and facilitates the development of long-standing professional relationships. From a career development perspective, a distributed model provides data scientists with greater opportunities to hone both their technical skills and their understanding of different aspects of drug discovery within their department. The risk of such a model is that these embedded data science teams can operate in silos, thus limiting crossfertilization of ideas and knowledge sharing. This can result in unnecessary duplication of efforts (and computational pipelines) across different departments within the same organization.

Connecting distributed data science teams is fundamental to effectively support the needs of individual departments while enabling an enterprise-wide stewardship of data science assets and talents. Data science teams should come together as part of a strong data science community with shared goals and accountability. Establishing an engaged network of data scientists across departments requires commitment from the different teams and can be enabled through regular scientific and social events, which facilitate knowledge sharing, create a sense of a diverse community with a common purpose, and foster collaborations across departments. Leaders of the different data science departments can facilitate this by forming a governance body that promotes such initiatives and implements a common strategy based on cross-departmental collaborations, unification of analytics solutions, and collaborative investment in talent development.

## Rule 6: Promote a culture of digital savvy across the organization

Drug discovery is a multifaceted science, and data scientists cannot achieve a digital transformation in isolation. Just as successful data scientists must gain a good understanding of the biological and experimental details behind the data they analyze, experimental scientists and clinicians must be engaged in the digital transformation by developing some level of digital proficiency and basic understanding of the mechanics underlying data science. Initiating company-wide education efforts will enable each scientist to make better use of novel data science technologies and to leverage their own data in the context of other internal or external datasets.

Experimental scientists are not expected to develop advanced computational or statistical skills, and computational scientists do not need to learn how to run experiments in the lab; rather, mutual exposure and understanding of each other’s discipline will ultimately pave the way for a more effective collaborative environment. Importantly, to fully unlock the potential of data generation and mining efforts, computational and experimental scientists should dedicate time to agree on experimental design best practices, with data scientists promoting and leading a question-based approach to data analysis. Ultimately, the aim is to remove communication barriers between computational and experimental scientists and develop hybrid scientists that can effectively use data to bridge the two disciplines.

## Rule 7: Embrace and deploy AI without hyping it

Together with the increased generation of large amounts of data, the other factor driving the current transformation in biomedical data science in the industry is the widespread availability of more advanced machine learning methods, including deep neural networks. The impact these algorithms are having within the healthcare business cannot be understated, particularly for applications to imaging data.

While biostatisticians, computational biologists, and chemists have been using machine learning methods extensively for decades with little fanfare, the impressive performance of deep learning methods across a number of tasks has garnered considerable attention from the media, which has fueled unprecedented interest in AI among experts and nonexperts alike. Within a short period of time, the data scientist became a fashionable profession in high demand [24], with AI at times being oversold at levels that risk creating unrealistic expectations.

It is important to highlight the impact that machine learning approaches are having across the drug discovery pipeline, from disease understanding and target identification to biomarker discovery and patient stratification strategies. At the same time, we must communicate equally effectively the caveats, biases, and limitations of such approaches and that harvesting insights from large, annotated datasets and contextualizing the findings are still limiting steps. Ultimately, we believe deep learning and AI are not panaceas that will solve all challenges in the pharmaceutical industry. Instead, we see machine learning as one of the tools, albeit a powerful one, in a wider arsenal of computational and statistical approaches that enable data scientists to gain mechanistic insights and drive real impact in our industry.

## Rule 8: Complement internal capabilities with strategic partnerships

Innovation is happening on a global scale and beyond the walls of any single institution. It is critical to develop an organizational model that systematically complements internal capabilities with external opportunities by developing a collaborative ecosystem across the pharmaceutical industry, technology providers, and academic centers.

Oftentimes, novel algorithms or computational approaches for a problem at hand are developed and made available by the external community. Given the intensive demand for data scientists on drug discovery projects, the cutting-edge methods developed, benchmarked, and published by academic researchers can be rapidly applied and, if necessary, adopted for industry-scale data. Indeed, pharmaceutical data scientists typically embrace a fast follower approach to fill gaps by investing in innovative external technologies or methodologies and bringing in high-quality and well-established tools and resources. Data solutions that are reproducible, unified, and aligned with external best practices are key for contextualization of internal data with the wealth of public data. Free open source software helps to ensure FAIR data principles because it reduces the likelihood that data becomes inaccessible or unusable due to an inability to continue running software that is closed-source or restrictively licensed. The use of free open source software and resources is already at the core of many current data solutions in drug discovery and development, but the pharmaceutical industry should in turn give back to the external data science community by publishing and making relevant data, tools, and benchmarks available (see [25] for an example).

Public–private partnerships such as the Innovative Medicines Initiative (IMI) [26] and the Accelerating Medicines Partnership (AMP) [27] provide excellent platforms for data scientists to collaborate, not only between industry and academia but also across the spectrum of biotechnology and pharmaceutical companies involved in these projects. Within the pharmaceutical industry, the Pistoia Alliance [28] promotes precompetitive, collaborative projects with a strong focus on data curation and analytics, while the European Molecular Biology Laboratory-European Bioinformatics Institute (EMBL-EBI)” Industry Programme [29] offers workshops and training opportunities across a wide range of biomedical topics at the interface of drug discovery and data science.

## Rule 9: Allocate sufficient and appropriate resources to data science teams

While automation of computational pipelines and reuse of code can significantly improve the turnaround time of data analyses, no two projects are identical, and a tailored approach is always required. A careful, high-quality analysis that can deliver true business impact takes time.

To achieve their goals, data science teams should therefore be resourced appropriately. Organizations should progressively increase the ratio of computational to experimental scientists in order to supplement and empower data generation with proper analytics. Within organizations, the optimal balance will vary by department, with technology and platform groups possibly needing a higher ratio of computational scientists compared to disease- and program-centric departments. As a rule of thumb, our recommendation is to employ at least 10% data scientists in research departments to enable biomedical data science at a scale that truly maximizes impact. Currently, we believe most pharmaceutical companies have invested below this level.

While we have used the term “data scientist” in its most broad sense, it is essential for organizations to fully embrace the diversity of roles required for the engineering, curation, integration, analysis, and mining of drug discovery data. Specialization and complementarity of roles are needed to ensure that talented data scientists contribute in an optimal capacity and reflect the complexity of designing, maintaining, and gaining insights from a large data infrastructure. Data engineers build and maintain the data storage and management systems. Data stewards capture and organize data and metadata into standard formats amenable to crossexperimental learning. Data wranglers process the raw data into a (re)usable form and integrate different data sources and modalities. Data analysts work closely with biologists, chemists, and clinicians to address specific scientific questions. Most data scientists will be able to cover many of these roles, but all will have specialties.

Finally, organizations should consider that, beyond hiring diverse teams of data scientists, appropriate financial investment in digital resources is a necessary part of the strategy. Data scientists cannot deliver maximal impact without additional investment in the datasets, software licenses, external collaborations, professional services, and hardware needed to perform the work. Appropriate resourcing and differentiation of data science functions is pivotal in sparking a digital transformation in the pharmaceutical industry by making digital technologies readily available to drug discovery teams, integrating unstructured and heterogeneous datasets across departments, and seizing external data opportunities that could be boons for specific projects.

## Rule 10: Invest in attracting and retaining talent

Ultimately, the success of any initiative depends on the people who lead and execute it, and realizing the full potential of data science in the pharmaceutical industry is no exception. Whereas data are among the most valuable assets of pharmaceutical companies, it follows that the people able to extract actionable knowledge from it are too. The modern data scientist, who combines computational modeling with domain knowledge and scientific storytelling skills (i.e., the ability to transfer knowledge by creating a narrative from the data), is highly sought after across a growing number of sectors, including academia and consulting, financial, and technological firms. Moreover, the digital transformation of healthcare is not only affecting the pharmaceutical industry; an increasing number of technology firms are investing in health divisions and recruiting data scientists with biomedical expertise and backgrounds.

In order for pharmaceutical companies to be at the forefront of biomedical innovation in the coming decades, it is essential that they focus on attracting and retaining the best talent available in data science. Historically, computational scientists have often been hired in the pharmaceutical industry using the same systems, metrics, and titles as experimental scientists. While this makes sense from the perspective of equalizing education level and years of experience, it simply does not reflect the higher demand for (and lower supply of) data scientists on the global job market. In addition, computational biologists, chemists, and biostatisticians can leverage their quantitative skills in other fields, such as the technology and financial industries. As the data scientist is a relatively new professional figure, it is therefore important that pharmaceutical organizations continue to refine and align to benchmarks from the global data science market.

It is equally important to provide data scientists with opportunities to develop and specialize. In particular, data scientists should be afforded career progression opportunities that increase the scope of their influence on the company’s strategy. Internal transfers of talent between teams and departments need to be encouraged as they provide data practitioners development and learning opportunities, while skills and knowledge are retained within the organization.

Finally, while extrinsic motivations such as salary, benefits, and promotions are critical to attract and retain talent, intrinsic motivations play an equally important, if not greater, role. Leaders have the responsibility to provide teams with the ability to develop their own ideas and drive projects autonomously, to specialize, to hone and ultimately master their core skills, and to inspire a sense of purpose that goes beyond the data analysis tasks at hand [30]. That is where the pharmaceutical industry has an undeniable advantage compared to other fields; as data scientists, we have the opportunity to contribute directly to the development of medicines that save lives and improve the quality of life of people around the world.

## Information on the authors

The authors of this article are all part of the Computational Sciences Council, a team of data science group leaders belonging to different departments at the Novartis Institutes for BioMedical Research. The mission of the Computational Sciences Council is to drive a digital transformation via a number of strategic and scientific initiatives, including empowerment of a large community of data scientists that acts locally and globally to organize social and scientific events, mentorship activities, and cross department onboarding programs.
